# Prognostic Value and Potential Regulatory Mechanism of H19 in Stomach Adenocarcinoma

**DOI:** 10.1155/2022/7702626

**Published:** 2022-08-31

**Authors:** Hongyuan Guo, Xu Guo, Yuanyuan Su, Minghui Liu, Xi Chen, Hao Zhu, Zheng Fu

**Affiliations:** ^1^Nanjing Drum Tower Hospital Center of Molecular Diagnostic and Therapy, State Key Laboratory of Pharmaceutical Biotechnology, Jiangsu Engineering Research Center for MicroRNA Biology and Biotechnology, NJU Advanced Institute of Life Sciences (NAILS), Institute of Artificial Intelligence Biomedicine, School of Life Sciences, Nanjing University, Nanjing, Jiangsu 210023, China; ^2^Chemistry and Biomedicine Innovation Center (ChemBIC), Nanjing University, Nanjing, Jiangsu 210023, China; ^3^Research Unit of Extracellular RNA, Chinese Academy of Medical Sciences, Nanjing, Jiangsu 210023, China; ^4^School of Life Science and Technology, China Pharmaceutical University, Nanjing, Jiangsu 210009, China; ^5^Department of Orthopedics, Affiliated Jianhu Hospital of Nantong University, No. 666 Nanhuan Road, Jianhu, Yancheng, Jiangsu 224700, China

## Abstract

The first lncRNA discovered, H19, has been found to participate in the regulation of diverse biological processes, including the pathogenesis of stomach adenocarcinoma. In addition to its oncogenic function in tumor formation, a high level of H19 in tumor tissues has also been reported to be an indicator for poor prognosis. However, although many previous works have investigated the level of H19 as an independent indicator for prognosis, the real value of H19 in predicting survival has rarely been evaluated. In this study, we established a prognostic model and nomogram for stomach adenocarcinoma by combining the expression level of H19 with traditional indices, which showed the value of H19 in predicting the survival rates of patients. In addition, we investigated the mechanism underlying the correlation of the H19 level in cancer tissue with poor prognosis in patients. Our results showed that H19 could function as ceRNA by sponging five miRNAs, which may promote the progression of cancer.

## 1. Introduction

Gastric cancer is a prominent cancer worldwide and was responsible for over 1,000,000 new cases and estimated 783,000 deaths in 2018, making it the third leading cause of cancer death [1]. A higher incidence of gastric cancer was observed in Eastern Europe, Eastern Asia, and South America [2]. Among gastric cancers, stomach adenocarcinoma (STAD) is the most common subtype and accounts for 95% of the total number of malignancies [3]. The overall survival (OS) rate of advanced STAD remains low, with a 5-year survival rate of approximately 30% [4]. The detection of the disease at an early stage and treatment with surgical resection remains to be the optimal choice for STAD like many other kinds of cancer [5,6]. For advanced STAD, cytotoxic chemotherapy remains the main first-line treatment strategy [7,8]. Although the addition of targeted therapy in later-line treatment was proven to be beneficial when compared with chemotherapy alone [9–11], therapeutic targets for advanced gastric cancer are exceedingly rare. Therefore, the discovery of new biomarkers is likely to brew new precision treatments for treatment of STAD in the future [12,13], and investigation into the mechanisms underlying STAD may become the key to finding novel approaches for prognosis improvement and drug development.

In recent years, long noncoding RNAs (lncRNAs) have attracted considerable attention for their role in regulating cancer-related processes such as carcinogenesis, recurrence, metastasis, and drug resistance [14–16]. However, the clinical value of lncRNAs in STAD is very incompletely understood. In this study, we used both bioinformatic and experimental approaches to identify lncRNAs with dramatically changed expression and found that H19 was dramatically upregulated in STAD tissues. Next, we analyzed the potential pathological mechanism of H19 and highlighted its interactions with five candidate miRNAs in MKN-45 cells. Additionally, we demonstrated that the interactions between H19 and these miRNAs could promote migration, invasion, and drug resistance of STAD cells and analyzed the potential downstream target genes of miRNAs. Furthermore, we discovered that H19 is an index for poor prognosis in STAD patients and functions as an important oncogenic ceRNA during the pathological process of STAD. Our findings may identify novel targets for drug development or predictive biomarkers for the prognosis of STAD patients.

## 2. Materials and Methods

### 2.1. Cell Culture

The human gastric cancer cell line MKN-45 was purchased from iCell Bioscience Inc, which was authenticated by short tandem repeats (STR) profiling and confirmed to be mycoplasma-free. MKN-45 cells were cultured in the RPMI-1640 medium (Invitrogen, CA, USA) supplemented with 10% fetal bovine serum (Gibco, Australia) and penicillin/streptomycin (Gibco, MA, USA) at 37°C in a 5% CO_2_ water-saturated atmosphere. 3.

### 2.2. Transfection of Gastric Adenocarcinoma Cells

MKN-45 cells (5 × 10^5^) were cultured in a 6-well plate. At 60% confluence, 6 *μ*g of each type of RNA oligo/plasmid was utilized to transfect MKN-45 cells using Lipofectamine 2000 transfection reagent (Invitrogen, CA, USA). After 24 hours, the cells were evaluated for successful transfection.

### 2.3. Cell Migration Assay

MKN-45 cells were cultured in 6-well plates at a density of 2 × 10^5^ cells/well. At 90% confluence, the cell layer was scratched with a sterile yellow 200 *μ*L pipette tip and then washed three times in PBS. Fresh RPMI-1640 medium containing 2% FBS was then added to the cells. Three random fields of view were selected and imaged using an inverted microscope.

### 2.4. Cell Invasion Assay

The cell invasion assay was performed in transwell chambers. The transwell chambers were placed in a 24-well plate, and each chamber contained an insert with an 8 *μ*m pore size polyethylene terephthalate membrane (Corning Life Sciences, MA, USA). The treated MKN-45 cells were resuspended and seeded in the upper chambers in a serum-free medium. Cells at a density of 5 × 10^4^ cells/well (in 200 *μ*L) were seeded in the upper transwell chambers, in which the membrane was coated with Matrigel (BD Biosciences, MA, USA) and 500 *μ*L of complete growth medium was added to the bottom chambers. The noninvaded cells in the upper chamber were removed with cotton swabs. Invaded cells on the bottom surface of the membrane were fixed, stained with crystal violet, and observed using a microscope.

### 2.5. Flow Cytometry

MKN-45 cells were washed twice in cold PBS and resuspended in Annexin V binding buffer at a concentration of 1 × 10^6^ cells/mL. Then, 100 *μ*L of the cell suspension (1 × 10^5^ cells) was incubated with 5 *μ*L of FITC-Annexin V and 5 *μ*L of propidium iodide using an Apoptosis Detection Kit (BD Biosciences, CA, USA). Then, 400 *μ*L of binding buffer was added. The flow cytometry was used to determine the apoptosis rate.

### 2.6. Quantitative Real-Time PCR (RT-qPCR)

The TRIzol reagent (Invitrogen, CA, USA) was used to extract the total RNA from the cells. One microgram of total RNA was reverse-transcribed into cDNA using HiScript III RT SuperMix for qPCR (+gDNA wiper) (R323-01, Vazyme). RT-qPCR was performed in a 20 *μ*L reaction volume. Quantitative real-time PCR was performed with ChamQ Universal SYBR qPCR Master Mix (Q711-02, Vazyme) in a LightCycler 96 instrument (Roche). The relative gene expression was normalized to GAPDH and calculated by the 2^−ΔΔCT^ method.

RT-qPCR for microRNAs was performed using a miRNA 1st Strand cDNA Synthesis Kit (MR101-01, Vazyme). Quantitative real-time PCR analysis was performed with miRNA Universal SYBR qPCR Master Mix (MQ101-01, Vazyme). The RT-qPCR was performed in a LightCycler 96 system (Roche). The primer sequences used for RT-qPCR are given in Table S2. All experiments were conducted in triplicate.

### 2.7. RNA Pulldown Assay

The RNA pulldown assay was performed as previously described. Briefly, biotin‐labeled RNAs (antisense RNAs) were transcribed using Biotin RNA Labeling Mix (Promega Corporation). Biotinylated anti-H19 probes (5′-CTGCTGTTCCGATGGTGTCTTTGATGTTGGGCTGATGAGGTCTGGTTCCT-3′) were dissolved in binding and washing buffer and incubated with streptavidin agarose resin (Thermo Fisher Scientific Inc.). Then, MKN-45 cell lysates were incubated with probe‐coated streptavidin beads and the precipitated RNAs were extracted using the TRIzol reagent. The samples were prepared for RT-qPCR analysis.

### 2.8. Luciferase Reporter Assay

The lncRNA H19 sequence was inserted into the pMIR-REPORT plasmid (Ambion, Austin, TX, USA). In the mutant H19 plasmid, the sequences binding to the seed sequence were mutated (Table S3) and inserted into the pMIR-REPORT plasmid. The HEK293 T cells were seeded in 24-well plates and transfected with 0.5 *µ*g of this plasmid, 0.25 *µ*g of *β*-galactosidase (*β*-gal) plasmid, and 50 pmol of the miRNA mimic or scrambled miRNA. *β*-Gal expression was used for normalization. After 24 h, cells were harvested and analyzed for luciferase activity using the luciferase assay kits (Promega, WI, USA).

### 2.9. Public Data

Data on STAD were downloaded from The Cancer Genome Atlas (TCGA; https://portal.gdc.cancer.gov/, project: TCGA-STAD). We downloaded the expression matrices and clinical information for 442 STAD samples and removed 37 samples of cystic, mucinous, and serous neoplasms. The human gastric tissue data were downloaded from the Genotype-Tissue Expression (GTEx; https://gtexportal.org/) database.

### 2.10. Pancancer Analysis

The expression level of the lncRNA H19 and the correlations between the lncRNA H19 expression level and the cancer outcomes across cancer types were downloaded from the TIMER (Tumor IMmune Estimation Resource; version 2) web resource (https://timer.comp-genomics.org/) using the “Gene_DE” module and the “Gene_outcome” module.

### 2.11. Analysis of Differentially Expressed Genes (DEGs)

A total of 379 patients included in the TCGA-STAD project were divided into an H19-high group and an H19-low group based on the median expression level of H19. DEGs between the two groups were identified using the DESeq2 package. The DEGs with log_2_FC ≥ 1 and *P* adj <0.05 were considered significant.

### 2.12. Enrichment Analyses

The DEGs were subjected to KEGG/GO (Kyoto Encyclopedia of Genes and Genomes/Gene Ontology) enrichment analyses and GSEA (gene set enrichment analysis) using the clusterProfiler package and the org. Hs. eg. db package.

### 2.13. Prognosis Prediction

First, we integrated the disease-specific survival (DSS) times of 379 STAD patients with their clinical information. Then, we calculated the correlations of these variables with the DSS time of STAD patients with a univariate Cox proportional hazards regression model. Risk-related factors with *P* < 0.1 were included as variables in the multivariate Cox proportional hazards regression analysis. Finally, we established a prognosis prediction model for STAD by constructing a nomogram based on the results of the multivariate Cox proportional hazards regression analysis. The sensitivity of the nomogram model was evaluated with time-dependent ROC analysis using the roc package, the accuracy was evaluated with a calibration plot using the rms package, and the predictive value was evaluated by DCA (decision curve analysis) using the stdca.R function (https://www.mskcc.org/departm-ents/epidemiology-biostatistics/biostatistics/decision-curve-analysis).

### 2.14. Target Prediction

We used the “Custom Prediction” module of the miRDB web interface (https://mirdb.org/) to predict the miRNAs that bind to H19 [17]. Then, we intersected the results of the miRDB prediction with the downregulated miRNAs in the STAD datasets GSE62254 and GSE15459 to obtain the target miRNAs of H19 in STAD. To predict the target genes of the H19-targeted miRNAs, we used the “Target Search” module of the miRDB web interface and the TargetScan Human online database (https://www.targetscan.org/vert_80/) [18]. We intersected the outputs of the miRDB and TargetScan predictions with the upregulated DEGs in TCGA-STAD to obtain the target genes of the predicted miRNAs in STAD. Finally, we calculated the correlation coefficients and significance between each target gene and H19 in STAD.

### 2.15. Statistical Analysis

All statistical analyses were performed using GraphPad Prism 8. Data were first checked for normal distribution and differences among groups were then compared by one-way ANOVA with Dunnett's test to correct for multiple comparisons. Data are shown as the means with error bars showing the SEMs. Significance was assumed for ^*∗*^*P* < 0.05, ^*∗∗*^*P* < 0.01, and ^*∗∗∗*^*P* < 0.005.

## 3. Results

### 3.1. Identification of Differentially Expressed lncRNAs in GC

**Figure 1:**
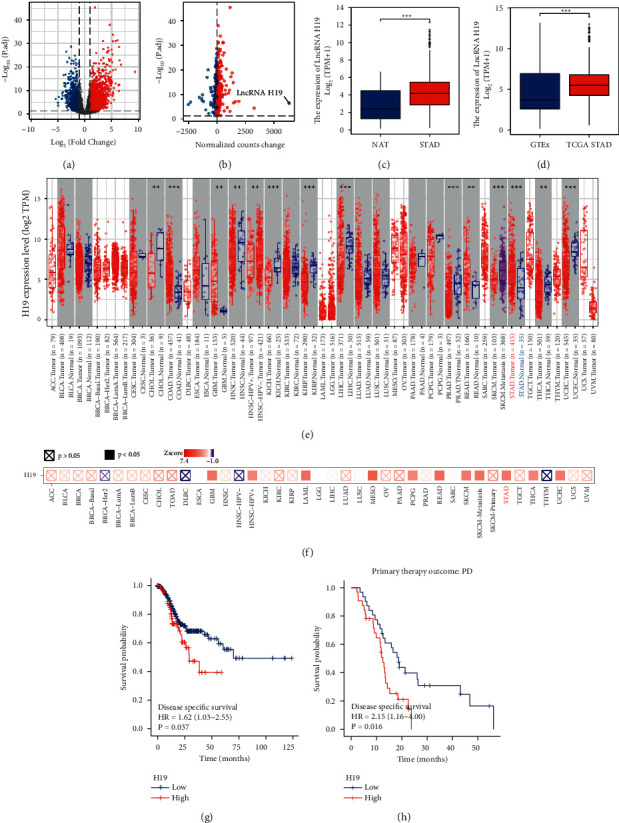
The expression of H19 in STAD and its correlation with prognosis. (a)The volcano plot of DELs between STAD and normal tissues. (b) Normalized transcript counts of significantly differentially expressed lncRNAs between STAD and normal tissues. (c) The expression level of lncRNA H19 in 379 STAD tissues and 26 normal tissues based on the TCGA database analysis. (d) The expression level of lncRNA H19 in 379 STAD tissues from the TCGA database and 174 normal tissues from the GTEx database. (e)The expression status of lncRNA H19 in different cancers and specific cancer subtypes analyzed with TIMER2. (f) The significant correlations of lncRNA H19 expression with outcomes across various cancer types visualized in the heatmap, which shows the normalized coefficient of lncRNA H19 in the Cox model. (g-h). The Kaplan–Meier curves for the DSS (c) and PD (d) of STAD patients stratified by the H19 expression level.

We downloaded STAD transcriptome data from the TCGA database and analyzed the differentially expressed lncRNAs with the DESeq2 package. The screening thresholds for differentially expressed lncRNAs (DELs) were |log_2_FC| > 1 and adjusted as *P* < 0.05, and the DELs are shown in volcano plots (Figure 1(a)). Among the 3669 differentially expressed lncRNAs, 2760 were upregulated, whereas 909 were downregulated. To better visualize the relative expression levels of DELs in tumor tissues, the changes in the normalized counts of these lncRNA transcripts were calculated (Figure 1(b)). The results showed that among all the lncRNAs, lncRNA H19 was strikingly overexpressed and significantly upregulated in STAD tissues. Next, we analyzed the level of H19 with transcriptome data from both the TCGA and GTEx databases (Figures 1(c) and 1(d)). The results showed that H19 was significantly overexpressed in STAD tissues in comparison with normal adjacent tissues (NATs). The TCGA dataset analysis also showed that the H19 level was significantly higher in STAD tissues. By analyzing tumors of different stages, we found that the expression level of H19 increased moderately with increasing tumor stage (Figure S1). By applying TIMER2, we analyzed the expression level of H19 across various cancer types, and the results also indicated that upregulation of H19 was obvious in STAD in comparison with other cancer subtypes (Figure 1(e)). Based on the TCGA data, we divided the patients with each cancer subtype into the high-expression and low-expression groups according to the level of H19 and analyzed the correlation of the H19 level with prognosis (Figure 1(f)). The results indicated a strong correlation of the H19 level with prognosis in STAD patients. Kaplan–Meier analyses of STAD patients were also performed with data from the TARGET database. Analysis of disease-specific survival (DSS) further showed that the survival rate in the H19-high group was significantly lower than that in the H19-low group (Figure 1(g)). Interestingly, in STAD patients that experienced progression after receiving adjuvant or neoadjuvant chemotherapy, the H19 level was an even more significant indicator for DSS, with a HR of 2.15 (1.16–4.00) (Figure 1(h)). These results indicated the universal overexpression of H19 in STAD and its correlation with poor prognosis in patients.

### 3.2. Inclusion of H19 in a Nomogram to Predict the Prognosis of GC

**Figure 2:**
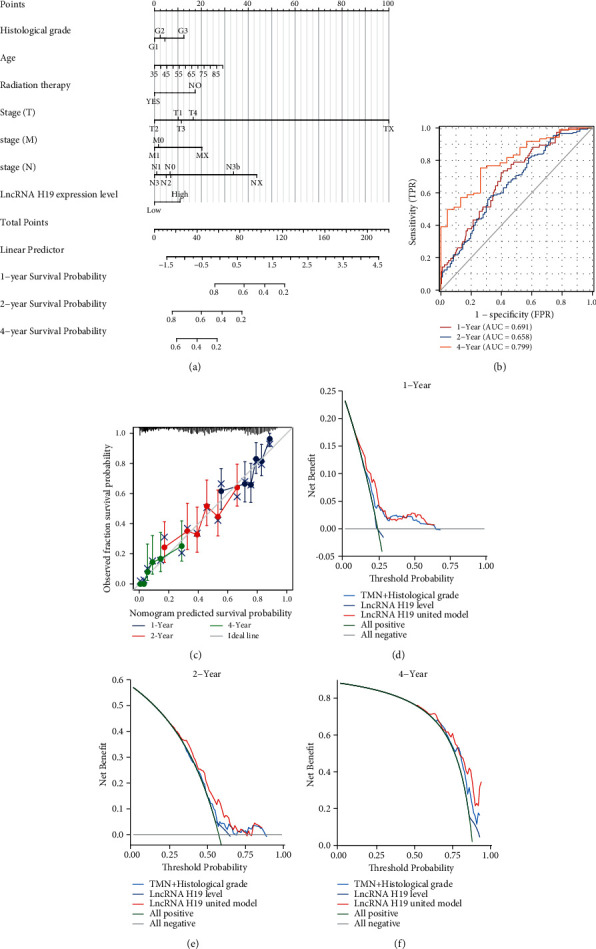
The prognostic value of H19 in STAD. (a) Prognostic nomogram for patients with STAD based on the H19 expression level. (b)Verification of the nomogram by time-dependent ROC curve analysis. (c) Calibration curves for predicting patient survival at each time point. (d–f) DCA curves showing the benefit gained from incorporation of H19 in predicting 1 (d), 2 (e), and 4 (f) year survival outcomes.

To further explore the prognostic value of H19, we developed a statistical model to predict the survival of STAD patients. The univariate Cox regression analysis was utilized to screen variables that correlated with prognosis using a threshold of *P* < 0.1. Univariate analysis indicated that TNM stage, age, histologic type, radiotherapy status, and H19 level were significantly associated with OS. After Cox multivariate regression analysis, four traditional clinical variables and the expression level of H19 achieved significance of *P* < 0.05 and were identified as prognostic factors (Table S1). Next, these factors were incorporated into nomograms for predicting the survival probability of STAD patients at 1, 2, and 4 years (Figure 2(a)). The nomogram identified TNM stage as having the largest contribution to prognosis, followed by age, histologic type, radiotherapy status, and H19 level. Each value for these variables was assigned a score on a point scale. By adding up the total score and locating it on the total point scale, we estimated the probability of survival at each time point. Then, the nomogram was validated internally using the TCGA dataset. Time-dependent ROC curves for the prognostic evaluation nomogram model were generated, and the AUCs of the nomogram for predicting 1, 2, and 4-year overall survival (OS) was 0.691, 0.658, and 0.799, respectively (Figure 2(b)). As shown in Figure 2(c), the calibration plots for prediction of 1, 2, and 4-year OS in both the training and validation sets indicated excellent agreement. These findings indicate that the nomogram including H19 can accurately predict OS in STAD patients. Furthermore, the DCA curves for the STAD survival assessment model with and without incorporation of the H19 expression level are shown. Although the model including the H19 expression level had little benefit for assessing the survival of STAD patients in the first year (Figure 2(d)), an obvious positive benefit for assessing two and four- year survival was shown (Figures 2(e) and 2(f)). Altogether, our results indicated that H19 may be a promising prognostic biomarker for survival in STAD patients.

### 3.3. Potential Biological Function of H19 in STAD

**Figure 3:**
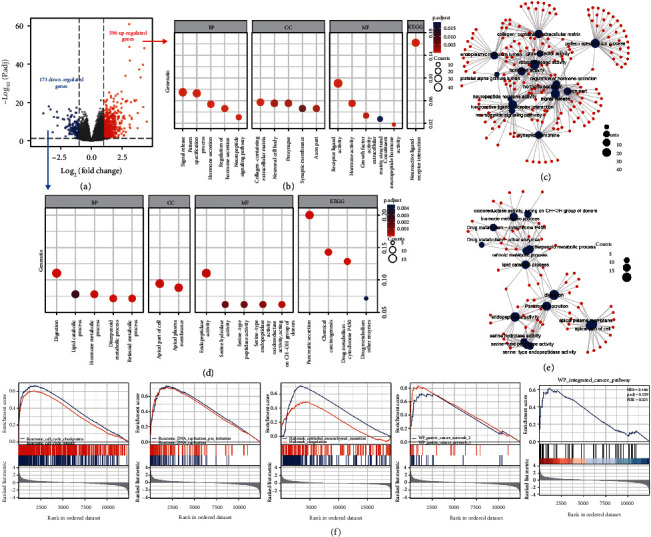
Differential expression analysis of samples with high and low H19 expression. (a) The volcano plot of DEGs between the H19-high and H19-low groups. (b–e) KEGG/GO enrichment analyses performed based on the 596 upregulated DEGs (b) and 173 downregulated DEGs (d); MF, BP, CC, and KEGG pathway analyses conducted and the corresponding networks based on the KEGG and GO analysis results constructed (c and e). (f) GSEA showing different gene expression patterns between the H19-high and H19-low groups.

Since an obvious correlation between the H19 level and the prognosis of STAD was shown by our results, we further investigated the potential underlying mechanism by investigating the biological function of H19 in STAD. According to the level of H19 expression, transcriptome data for STAD samples from TCGA were used to divide the corresponding patients into an H19-high group and an H19-low group according to the median expression level of H19. Then, the differentially expressed genes between these two groups were analyzed with the DESeq2 package with a threshold of |log_2_FC| > 1 and adjusted *P* < 0.05. As shown in the volcano plots in Figure 3(a), 596 genes were upregulated, whereas 173 genes were downregulated. Next, we performed KEGG and GO enrichment analyses, and the corresponding network diagrams are shown (Figures 3(b)–3(e)). As shown in the results, the biological processes “signal release,” “collagen-containing extracellular matrix,” “receptor ligand activity,” and neuroactive ligand-receptor investigation' were enhanced in the H19-high group, while “digestion,” “apical part of cell,” “endopeptidase activity,” and “pancreatic secretion” were suppressed. GSEA showed different gene expression patterns between the H19-high and H19-low groups (Figure 3(f)). The expression levels of genes related to the cell cycle, DNA replication, EMT, GC, and cancer pathways were positively correlated with the expression level of H19. All these pathways are related to the proliferation (cell cycle and DNA replication), invasion or migration (EMT), or maintenance (GC and cancer pathways) of malignant GC cells. These results reflected the biological effect of H19 on the transcriptome profile and justified the correlation between the H19 level and poor prognosis in STAD.

### 3.4. The Potential Function of H19 as a ceRNA in STAD

**Figure 4:**
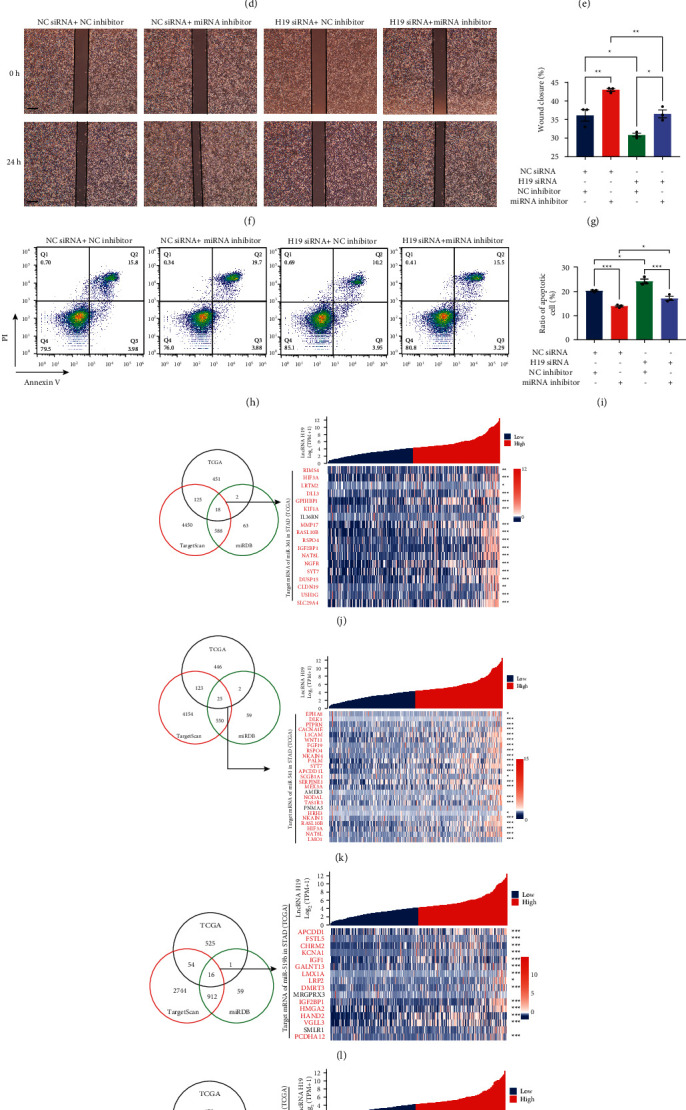
H19 promoted migration, invasion, and survival of STAD cells by binding to five miRNAs. (a) Bioinformatics analysis of 9 miRNAs potentially binding to lncRNA H19. (b) RNA pulldown assay verified the binding of 5 of the 9 miRNAs to lncRNA H19 (*n* = 3). (c) Relative luciferase activity determined to verify the binding of 5 miRNAs to H19 (*n* = 3). (d) Scratch assay showing the migration of MKN-45 cells with H19 knockdown or miRNA inhibition (*n* = 3). (e) Quantitative analysis of the wound closure rate (*n* = 3). (f) Transwell assays showing the invasion of MKN-45 cells after H19 knockdown or miRNA inhibition (*n* = 3). (g) Quantitative analysis of the number of migrated cells (*n* = 3). (h) Flow cytometric analysis of apoptosis induced by gemcitabine in MKN-45 cells transfected with H19 siRNA (*n* = 3). (i) Quantitative analysis of the percentage of apoptotic cells (*n* = 3). (j-n) Analysis of potential downstream genes regulated by five miRNAs binding to H19.

According to the normalized counts of H19 transcripts in STAD tissues (Figure 1(b)), the expression level of H19 in STAD tissues should be strikingly high, indicating that it is likely to act as a molecular sponge for miRNAs. Bioinformatics analysis revealed that lncRNA H19 has putative miRNA recognition sequences for 9 miRNAs (Figure 4(a)). The minimum free energy of hybridization between each miRNA and H19 was calculated by RNAhybrid. The predicted interactions between these miRNAs and the target sites in H19 are shown in Figure S2, and all of the minimum free energies of hybridization were less than −25 kcal/mol. The RNA pulldown assay showed that the lncRNA H19 binds to miR-361, miR-519a, miR-541a, miR-516b, and miR-193a in the MKN-45 gastric cancer cell line (Figures 4(b) and S3). The knockdown of H19 expression in MKN-45 cells significantly increased the cellular levels of all 5 candidate miRNAs (Figure S4). Next, we designed luciferase reporter plasmids containing H19 with wild-type (WT) or mutant (MUT) miRNA binding sites to verify the binding capacity between H19 and the candidate miRNAs (Figure 4(c)). The luciferase assay results showed that the mimics of all five miRNAs significantly inhibited the activity of the WT luciferase reporter but not the MUT reporter. These results indicated that H19 can directly bind to these miRNAs at the predicted binding sites in gastric cancer cells.

To further validate the biological function of H19 as a ceRNA, we explored whether modulating H19/miRNA regulation affects the characteristics of gastric cancer cells. First, we investigated the influence of the H19/miRNA interactions on the invasion ability (Figures 4(d) and 4(e)). The transwell assay showed that knockdown of H19 significantly inhibited MKN-45 cell invasion, while inhibition of these miRNAs significantly enhanced the invasion ability. When H19 and these miRNAs were inhibited simultaneously, the effects canceled each other out. Similar results were also observed in the scratch assay to assess the migration ability (Figures 4(f) and 4(g)). The knockdown of H19 attenuated the migration ability of MKN-45 cells, while inhibition of miRNAs promoted it. Simultaneous inhibition of H19 and the miRNAs resulted in almost complete elimination of the independent effects. Next, we evaluated the effects of H19/miRNA interactions on drug resistance in gastric cancer cells. Gemcitabine, a commonly used chemotherapeutic drug for treating STAD, was used to induce apoptosis in MKN-45 cells. The transfection of H19 siRNA significantly increased the apoptosis of MKN-45 cells and depletion of the miRNAs with inhibitors reduced apoptosis (Figure 4(h) and 4(i)). After cotransfection of H19 siRNA and miRNA inhibitors, apoptosis remained at the baseline level. These experiments covered the invasion, migration, and drug resistance properties of cancer cells and proved that H19 performed an oncogenic function by sponging these five miRNAs, which may result in the poor prognosis of STAD patients with a high H19 level. To further identify the potential target genes of these five miRNAs, we predicted their binding sites for the 3′UTRs of mRNAs in the human transcriptome with both TargetScan and miRDB (Figures 4(j)–4(n)). Then, we intersected the prediction results with the downregulated genes in H19-high tissues compared with H19-low tissues and obtained the potential genes that are affected by the overexpression of H19 in gastric cancer tissues through its action as a miRNA sponge. The lncRNA–miRNA–mRNA network was constructed to demonstrate the regulatory relationships between the miRNAs and key genes, as well as the enriched pathways and annotations of the key genes (Figure S5).

## 4. Discussion

In recent years, the importance of noncoding RNAs as clinical biomarkers for cancer diagnosis and prognosis has been widely recognized [19–21]. Among the numerous noncoding RNAs, lncRNA H19 is one of the most frequently studied. Chen et al. measured the expression level of H19 in 128 pairs of STAD and adjacent normal tissues and generated ROC curves and Kaplan–Meier curves to prove its diagnostic or prognostic value [22]. Other studies have also supported H19 as a diagnostic biomarker for STAD [23,24]. However, most of these studies investigated lncRNAs as independent novel biomarkers and did not combine them with traditional variables for diagnosis. Here, we showed the real value of H19 by developing a model including relevant clinical variables for STAD prognosis. The contribution of H19 in comparison with other indices was clearly shown in the nomogram. In addition, since all the data included in the model are publicly available, the model is unbiased compared with most studies conducted with a limited cohort of samples. Our results demonstrated that H19 should be used in combination with traditional clinical indices such as TNM stage or histological grade to predict STAD prognosis. The incorporation of H19 into the model showed clear benefit for predicting the survival prognosis at 2 and 4 years and did not decrease the performance of the model in predicting 1-year survival. To our knowledge, this is the first study to integrate the clinical factors and H19 to construct a nomogram to predict the prognosis of STAD patients.

Second, we also investigated the potential biological mechanism underlying the prognostic value of H19. Through analysis of DEGs in H19-high STAD samples compared with H19-low STAD samples, we highlighted the possible biological processes and gene sets related to the poor prognosis of STAD patients. The gene sets related to the invasion, migration, and malignancy of STAD were found to be differentially regulated by H19 upregulation. These results were also supported by our in vitro experiments. Our results showed that H19 influences classical tumorigenic processes such as invasion, migration, and drug resistance. We validated the ceRNA function of H19 in MKN-45 cells and identified a panel of five miRNAs that directly bind to H19 in MKN-45 cells. Potential downstream targets were identified by combining the miRNA target prediction tools and analysis of DEGs in the TCGA database, which provided insights for further studies.

Overall, our study verified the prognostic value of H19 in STAD and established a nomogram for predicting the survival rate of STAD patients. The validation of the nomogram demonstrated the contribution of the H19 level to increasing the accuracy of the prediction model incorporating only traditional clinical indices. We also highlighted the mechanism underlying the positive correlation between the H19 level and poor prognosis in STAD patients. Our results indicated the interactions between H19 and five miRNAs and identified candidate downstream target genes for further study of the role of H19 in STAD pathogenesis.

## Data Availability

The data used to support this study are available from the corresponding author upon request.

## Supplementary Materials

Supplementary Materials Figure S1. Relative H19 level in T1–T4 stages tumor tissues from TCGA database. Figure S2. Schematic description of the duplex formed by the binding site of lncRNA H19 and miRNAs. Figure S3. The pulldown efficacy of lncRNA H19 with biotinylated lncRNA H19 probes. Figure S4. The knockdown efficiency of lncRNA H19 in MKN-45 cells. Figure S5. The ceRNA network showing the relationship between lncRNA H19, miRNAs, and downstream target genes. Table S1. Univariate and multivariate analyses Cox proportional hazards regression analysis. Table S2. The primer sequences of RT-qPCR. Table S3. The binding site sequence and mutant sequence used for constructing luciferase reporter gene plasmids.
