# Supplementary material for: Prognostic Value and Potential Regulatory Mechanism of H19 in Stomach Adenocarcinoma
Source: J Oncol. 2022 Aug 31;2022:7702626. doi: 10.1155/2022/7702626 (PMC9452980; doi:10.1155/2022/7702626)
Supplement: Supplementary Materials — Figure S1. Relative H19 level in T1–T4 stages tumor tissues from TCGA database. Figure S2. Schematic description of the duplex formed by the binding site of lncRNA H19 and miRNAs. Figure S3. The pulldown efficacy of lncRNA H19 with biotinylated lncRNA H19 probes. Figure S4. The knockdown efficiency of lncRNA H19 in MKN-45 cells. Figure S5. The ceRNA network showing the relationship between lncRNA H19, miRNAs, and downstream target genes. Table S1. Univariate and multivariate analyses Cox proportional hazards regression analysis. Table S2. The primer sequences of RT-qPCR. Table S3. The binding site sequence and mutant sequence used for constructing luciferase reporter gene plasmids. [file 7702626.f1.docx]

**Supplementary information for**

**Prognostic value and potential regulatory mechanism of H19 in stomach adenocarcinoma**

**(Hongyuan Guo *et al.*)**


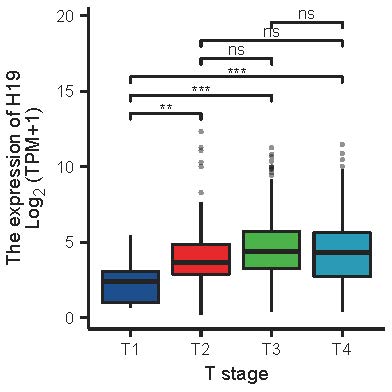


Fig S1. Relative H19 level in T1-T4 stages tumour tissues from TCGA database.


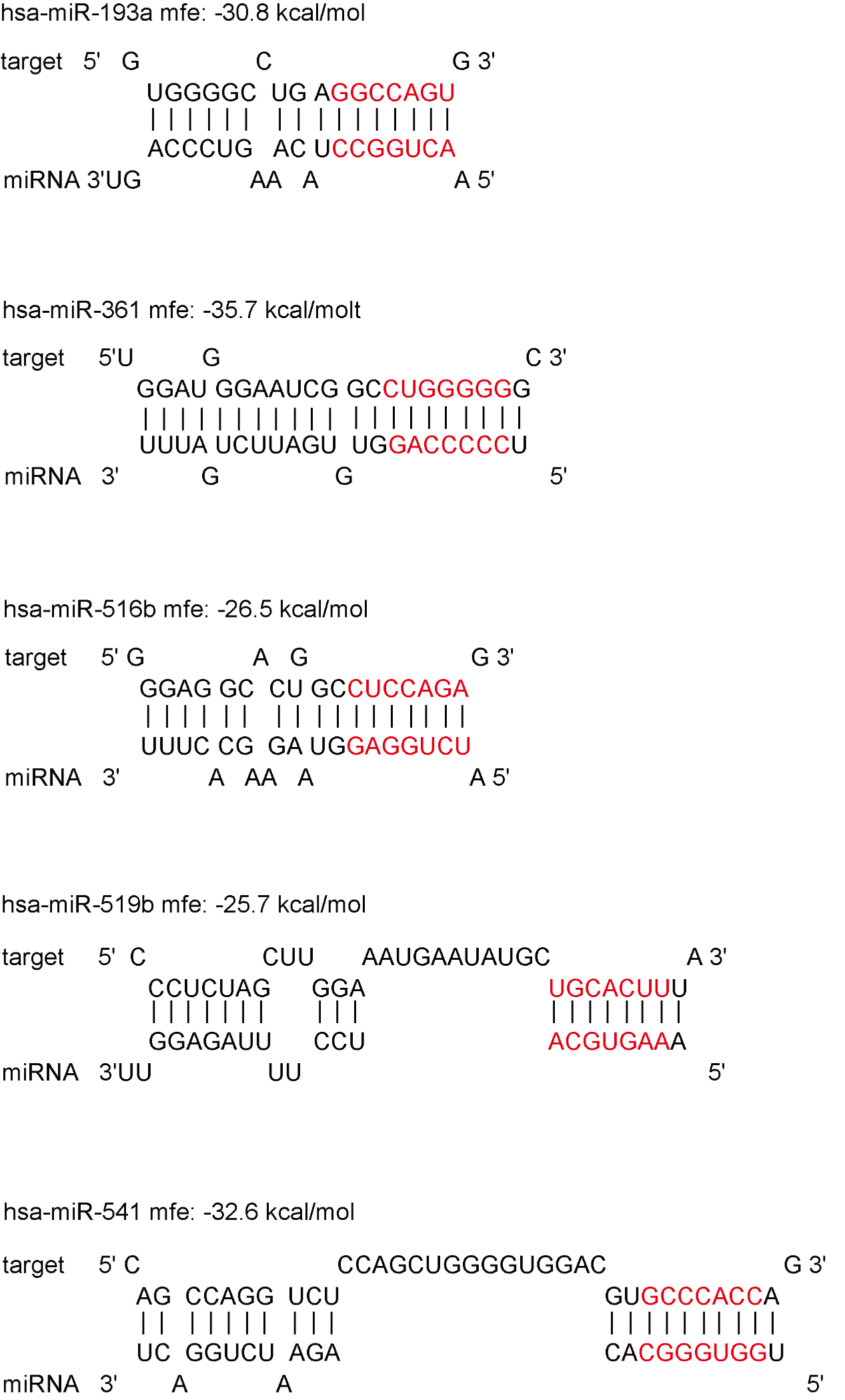


Fig S2. Schematic description of the duplex formed by the binding site of lncRNA H19 and miRNAs.


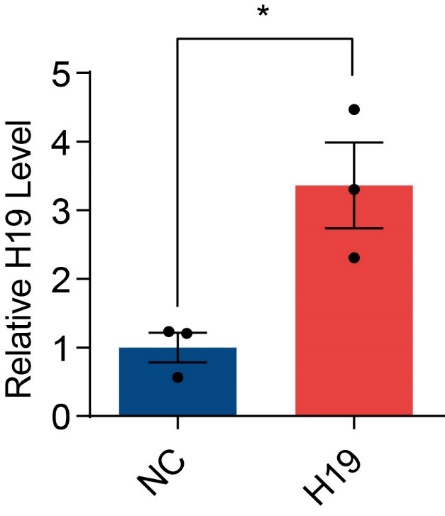


Fig S3. The pulldown efficacy of lncRNA H19 with biotinylated lncRNA H19 probes.


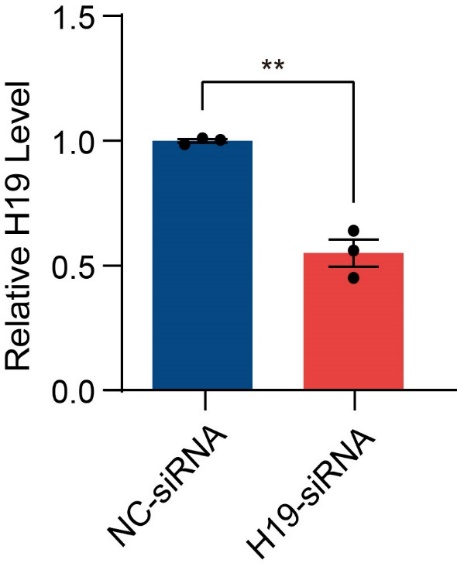


Fig S4. The knockdown efficiency of lncRNA H19 in MKN-45 cells.


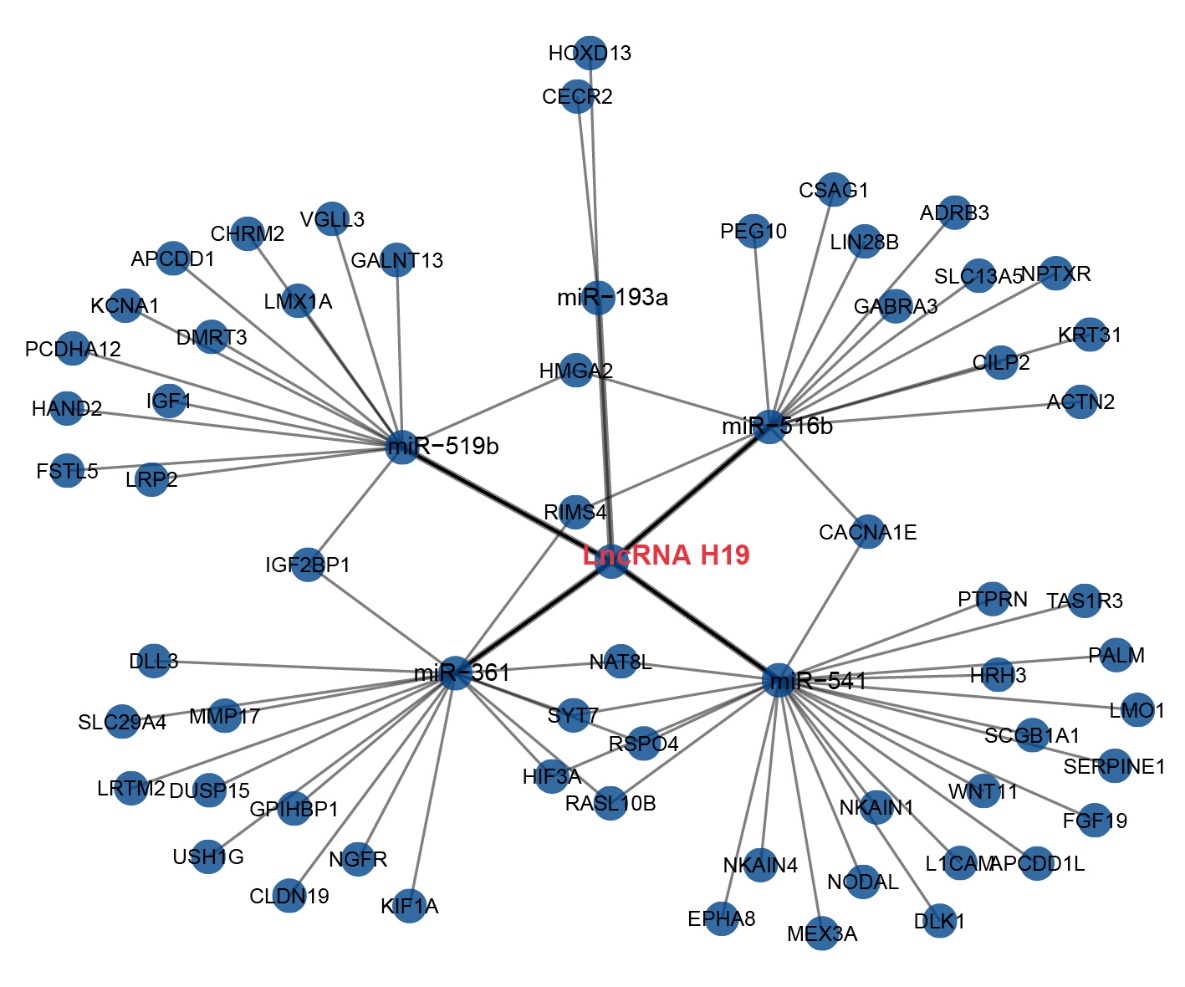


Fig S5. The ceRNA network showing the relationship between lncRNA H19, miRNAs, and downstream target genes.

| Characteristics | Total(N) | Univariate analysis | |  | Multivariate analysis | |
| --- | --- | --- | --- | --- | --- | --- |
|  |  | Hazard ratio (95% CI) | P value |  | Hazard ratio (95% CI) | P value |
| AJCC pathologic tumorstage | 332 |  |  |  |  |  |
| Stage I | 51 | Reference |  |  |  |  |
| Stage III | 141 | 1.075 (0.751-1.539) | 0.693 |  |  |  |
| Stage IV | 33 | 0.938 (0.546-1.609) | 0.815 |  |  |  |
| Stage II | 107 | 1.346 (0.926-1.957) | 0.119 |  |  |  |
| Histological grade | 332 |  |  |  |  |  |
| G2 | 116 | Reference |  |  |  |  |
| G3 | 200 | 1.406 (1.071-1.847) | **0.014** |  | 1.357 (1.014-1.814) | **0.040** |
| G1 | 8 | 1.033 (0.450-2.369) | 0.939 |  | 0.929 (0.394-2.192) | 0.866 |
| GX | 8 | 1.106 (0.481-2.541) | 0.812 |  | 1.062 (0.451-2.505) | 0.890 |
| Age | 332 | 1.013 (1.000-1.025) | **0.047** |  | 1.016 (1.002-1.030) | **0.021** |
| Sex | 332 |  |  |  |  |  |
| MALE | 215 | Reference |  |  |  |  |
| FEMALE | 117 | 1.055 (0.814-1.368) | 0.684 |  |  |  |
| Race | 332 |  |  |  |  |  |
| WHITE | 227 | Reference |  |  |  |  |
| ASIAN | 93 | 1.341 (1.016-1.769) | **0.038** |  |  |  |
| BLACK OR AFRICAN AMERICAN | 11 | 0.719 (0.318-1.625) | 0.428 |  |  |  |
| NATIVE HAWAIIAN OR OTHER PACIFIC ISLANDER | 1 | 0.000 (0.000-Inf) | 0.993 |  |  |  |
| Radiation therapy | 332 |  |  |  |  |  |
| NO | 271 | Reference |  |  |  |  |
| YES | 61 | 0.531 (0.385-0.732) | **<0.001** |  | 0.592 (0.416-0.842) | **0.004** |
| Stage(T) | 332 |  |  |  |  |  |
| T1 | 14 | Reference |  |  |  |  |
| T3 | 157 | 1.029 (0.577-1.833) | 0.923 |  | 1.041 (0.563-1.926) | 0.897 |
| T2 | 69 | 0.879 (0.477-1.618) | 0.678 |  | 0.737 (0.389-1.394) | 0.347 |
| T4 | 90 | 1.222 (0.671-2.225) | 0.513 |  | 1.215 (0.630-2.343) | 0.560 |
| TX | 2 | 31.516 (3.609-275.233) | **0.002** |  | 15.109 (1.569-145.520) | **0.019** |
| stage(M) | 332 |  |  |  |  |  |
| M0 | 298 | Reference |  |  |  |  |
| M1 | 18 | 0.916 (0.499-1.681) | 0.777 |  | 0.949 (0.490-1.842) | 0.878 |
| MX | 16 | 1.845 (1.052-3.236) | **0.033** |  | 1.751 (0.963-3.183) | 0.066 |
| stage(N) | 332 |  |  |  |  |  |
| N0 | 98 | Reference |  |  |  |  |
| N2 | 69 | 0.884 (0.615-1.270) | 0.505 |  | 0.950 (0.636-1.420) | 0.803 |
| N1 | 87 | 0.770 (0.555-1.070) | 0.120 |  | 0.841 (0.587-1.206) | 0.347 |
| N3 | 21 | 0.589 (0.295-1.174) | 0.133 |  | 0.817 (0.398-1.676) | 0.582 |
| N3a | 39 | 1.090 (0.708-1.680) | 0.695 |  | 1.009 (0.633-1.608) | 0.971 |
| NX | 12 | 2.739 (1.452-5.165) | **0.002** |  | 3.053 (1.514-6.157) | **0.002** |
| N3b | 6 | 1.844 (0.745-4.567) | 0.186 |  | 2.264 (0.884-5.795) | 0.088 |
| LncRNA H19 expression level | 332 |  |  |  |  |  |
| Low | 246 | Reference |  |  |  |  |
| High | 86 | 1.347 (1.001-1.814) | **0.049** |  | 1.392 (1.024-1.891) | **0.035** |

Table S1. Univariable and Multivariate Analysis Cox Proportional Hazards Regression Analysis

| miRNA | RT primer | PCR F primer | PCR R primer |
| --- | --- | --- | --- |
| hsa-miR-193a | GTCGTATCCAGTGCAGGGTCCGAGGTATTCGCACTGGATACGACACTGGG | CGCGAACTGGCCTACAAAGT | AGTGCAGGGTCCGAGGTATT |
| hsa-miR-361 | GTCGTATCCAGTGCAGGGTCCGAGGTATTCGCACTGGATACGACAAATCA | CGTCCCCCAGGTGTGATTC | AGTGCAGGGTCCGAGGTATT |
| hsa-miR-516b | GTCGTATCCAGTGCAGGGTCCGAGGTATTCGCACTGGATACGACAAAGTG | GCGCGATCTGGAGGTAAGAAG | AGTGCAGGGTCCGAGGTATT |
| hsa-miR-519b | GTCGTATCCAGTGCAGGGTCCGAGGTATTCGCACTGGATACGACAACCTC | CGCGAAAGTGCATCCTTTTA | AGTGCAGGGTCCGAGGTATT |
| hsa-miR-541 | GTCGTATCCAGTGCAGGGTCCGAGGTATTCGCACTGGATACGACAGTCCA | GCGTGGTGGGCACAGAATC | AGTGCAGGGTCCGAGGTATT |
| H19 | —— | ACTCAGGAATCGGCTCTGGAA | CTGCTGTTCCGATGGTGTCTT |

Table S2. The primer sequences of RT-qPCR

| miRNA | Seed sequence | Seed sequence (mut) |
| --- | --- | --- |
| hsa-miR-193a | GUCCCAG | CAGGGUC |
| hsa-miR-361 | UCUGAUU | AGACUAA |
| hsa-miR-516b | AGCACUU | UCGUGAA |
| hsa-miR-519b | UAGAGGU | AUCUCCA |
| hsa-miR-541 | UCUGGAC | AGACCUG |

Table S3. The binding site sequence and mutant sequence used for constructing luciferase reporter gene plasmids.
